# Reconciling competing priorities in commissioning: the future of bone densitometry service for North Wales

**DOI:** 10.1186/1478-7547-5-1

**Published:** 2007-01-18

**Authors:** Robert L Atenstaedt, Sandra Payne, Richard Roberts, Ian Russell, Daphne Russell, Rhiannon Tudor Edwards

**Affiliations:** 1 National Public Health Service for Wales (North Wales Region), Mold CH7 1PZ, UK; 2 Institute for Medical & Social Care Research, University of Wales, Bangor LL57 2PX, UK; 3 Centre for Economics and Policy in Health, University of Wales, Bangor LL57 1UT, UK

## Abstract

**Background:**

Osteoporosis creates brittle bones susceptible to fracture, with resulting high levels of morbidity and mortality. Poor access to bone densitometry services for the residents of North Wales led to the Welsh Assembly Government offering capital to purchase a dual-energy X-ray absorptiometry (DXA) scanner, used to diagnose osteoporosis, for the region. The commissioning question for the six Local Health Boards across North Wales was where to site the new scanner. This decision needed to reflect current inequalities in access to services and concerns over inappropriate prescribing relative to Welsh norms.

**Methods:**

Epidemiological, corporate and comparative healthcare needs assessments were performed. In addition, two cross-sectional surveys were conducted to determine the views of general practices and users of bone densitometry services resident in North Wales. An option appraisal and sensitivity analysis of 13 costed options for DXA scanning was conducted.

**Results:**

We estimated that only 31% of the people in North Wales who met national guidelines were receiving DXA scans. There was definite inequity of access to the current service provided by area of residence. There was also evidence of inequity of access by age and sex. The most suitable option identified in the option appraisal was a bone densitometry service based in the central location of Llandudno.

**Conclusion:**

The assessment identified significant unmet need for DXA scanning. A recommendation was made to improve access through the introduction of a new bone densitometry service based at Llandudno. This would double scanning provision provided and reduce travel costs and time for many North Wales residents. This recommendation was adopted by a joint commissioning group established by the six Local Health Boards in North Wales at the end of 2004 – evidence based commissioning in practice.

## Background

Osteoporosis is a disease which creates brittle bones susceptible to fracture. The World Health Organisation (WHO) defines osteoporosis in terms of bone density [1]. A woman has osteoporosis when her bone density falls more than 2.5 standard deviations below the young adult mean value. BMD measurements at the hip have the highest predictive value for hip fracture, with the WHO diagnostic criteria having a specificity of 80% and a sensitivity of 30% [2].

A diagnosis of osteoporosis might be suggested by the presence of risk factors, but needs to be confirmed using bone densitometry. Although there are other methods available for measuring bone density such as quantitative ultrasound (QUS) and peripheral dual-energy X-ray absorptiometry (pDXA), central DXA is recognised as the gold-standard diagnostic technique because of its flexibility to assess different skeletal sites, its high reproducibility and its low radiation dose [3]. QUS and pDXA measure sites on the peripheral skeleton (usually heel and/or forearm) while central DXA measured sites on the central skeleton (e.g. spine and hip).

Treatment programmes with prior bone density measurement are far more cost effective than those without, especially for relatively expensive medications such as bisphosphonates (which will increasingly be used for osteoporosis) [4]. The costs per QALY (Quality Adjusted Life Year) gained and per year of life gained for osteoporosis treatment with prior bone density measurement in those found to have high risk of fracture compare favourably with the treatment of hypertension and hypercholesterolaemia [4]. There is evidence that screening by pDXA or QUS before referral to central DXA is not cost-effective [4,5]. A case-finding approach based on risk factors is recommended, rather than universal screening, which would result in over-treatment of patients without increased fracture risks [6].

**Figure 1:**
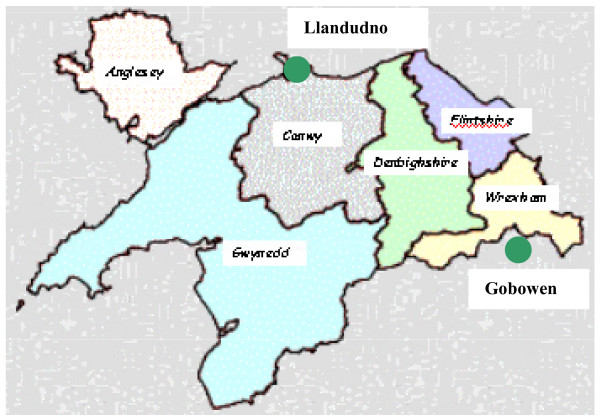
**Map of North Wales with relevant sites**. The figure shows a map of North Wales with the location of the current service provider in Gobowen, Shropshire and the proposed new service location in Llandudno. The coloured areas represent the 6 LHBs.

The current bone densitometry service provision to North Wales residents is an open access static central DXA service at Robert Jones and Agnes Hunt NHS Trust, a specialist orthopaedic hospital located outside the eastern border of the region in Gobowen, Shropshire. This can entail a four hour round trip for some residents. Figure 1 shows a map of North Wales with relevant sites.

Poor access to this service led to the Welsh Assembly Government (WAG) making the offer of capital to purchase a DXA scanner for the region. Six Local Health Boards (LHBs) across North Wales, similar to Primary Care Trusts (PCTs) in England, are responsible for commissioning primary and secondary NHS services for the population of North Wales. These bodies had to decide where to site this new scanner.

Healthcare needs assessment is defined as 'the systematic approach to ensuring that the health service uses its resources to improve the health of the population in the most efficient way'[7]. It is usually accepted that this occurs within finite resources [8]. If healthcare needs are to be identified, then an effective intervention should be available to meet these needs and improve health. There will be no benefit from an intervention that is not effective [7]. Healthcare needs assessment must balance clinical, ethical, and economic considerations of need – that is, what services should be provided, how and where should they be provided and on what basis should patients have access to these services? The approach also provides a method of monitoring and promoting equity in the provision and use of health services and addressing inequalities in health [9,10]. In this study, healthcare needs will be taken to be the population's ability to benefit from health care i.e. preventive, diagnostic or treatment services [11]. The three complementary approaches to health needs assessment are epidemiological, corporate and comparative.

### Aim

The aim of the six LHBs was, through evidence based commissioning, to improve the equity of access to effective and efficient bone densitometry services for the 650,0000 residents of North Wales.

In order to provide evidence to support this commissioning aim, our research objectives were as follows:

1. to estimate the burden of osteoporosis and related fractures in North Wales

2. to review the clinical effectiveness and cost-effectiveness of DXA scanning for the diagnosis of osteoporosis

3. to estimate the need for bone densitometry within North Wales

4. to review the current DXA service provided to the residents of North Wales

5. to develop an evidence based commissioning plan to address any unmet need and inequity of access to bone densitometry services.

## Methods

The six LHBs in North Wales commissioned the National Public Health Service for Wales and the Institute of Medical and Social Care Research (IMSCaR) at the University of Wales Bangor, to carry out a needs assessment and provide information for the planning of a bone densitometry service for North Wales.

Epidemiological, corporate and comparative healthcare needs assessments were performed from an NHS perspective.

### Epidemiological needs assessment

The key components in epidemiological needs assessment are: a description of the existing services, the prevalence and incidence of disease and effectiveness and cost-effectiveness of interventions and associated services. The number of people with osteoporosis in North Wales was estimated by applying prevalence estimates for osteoporosis of the hip (using WHO definitions) in men and women aged 50 years or older in England and Wales [12] to population estimates.

### Corporate needs assessment

The corporate approach has the advantage of allowing local circumstances to be taken into account and is based on the views of interested groups e.g. health care professionals, pressure groups and the public. In this study, only GPs and service users were consulted because of resource constraints. To this end, two cross-sectional surveys were conducted during 2003/4 to determine the views of referring general practices and DXA service users resident in North Wales after ethical approval had been received from the Local Research Ethics Committees across North Wales. These are as follows:

#### Professional views

The aim of the survey of general practices was to review their satisfaction with the current bone densitometry service, accessibility issues and preferences for future services in order to inform planning decisions. The sampling frame, a list of all GP practices in North Wales, was compiled from the web-sites of the 6 LHBs in North Wales. There were a total of 121 listed. A 100% sample was selected as it was felt important that all practices contribute to planning decisions. There were no specific inclusion or exclusion criteria. A postal questionnaire for self-completion by practice managers was constructed using information gathered from the literature and from advice provided by colleagues. The questionnaire was in two parts. The first part was designed to elicit information on whether the GP practice had referred patients to the static DXA scanner in Gobowen in the past 12 months. If they had, they were asked to rate their satisfaction with the service that they had received. They were then asked whether they had had any patients in the previous month who had needed a DXA scan but had refused or been unable to travel to the existing service because of travel distance. The second part of the questionnaire sought to gather information on the opinion of the GP practice on future service options for bone densitometry. The practice was given four options and they were asked to rank these in order of preference and to indicate any that they would not use. The options were 'existing service provided in Gobowen', 'new DXA service based at Llandudno Hospital', 'new DXA service based elsewhere in North Wales' and 'new mobile DXA service covering all of North Wales'. For option 3, the practice was asked where they would like this new service to be located. After piloting the questionnaire at a small number of practices in North Wales, the final version was sent to practice managers, accompanied by a covering letter. This explained the purpose of the study, stated that permission had been obtained from the relevant ethical committees and gave them a contact number for further information. Also included with the questionnaire was a self-addressed Freepost envelope for return of completed questionnaires. Participants were initially given a two-week return date. If they had not responded in another two weeks, they were given a telephone reminder and the opportunity to complete the questionnaire over the phone. The results of the questionnaire were then collated in Excel (spreadsheet software from Microsoft) and analysed using SPSS (Statistical Package for the Social Sciences).

#### User views

The aim of the user survey was to elicit the views of residents of North Wales who had recently attended the Gobowen service for a DXA scan, including their satisfaction with the current service, accessibility issues and preferences for the future service. Contact details for the last 200 patients from North Wales who had attended Gobowen for a scan were obtained. This sample was then stratified by LHB and weighted in proportion to the number of people in the LHB area who are estimated to have osteoporosis. The total sample selected was 178. A questionnaire was planned and designed with colleagues including those who had done a previous service evaluation of telemedicine activity [13]. Each client was given an identifying number which was used from that point forward on the questionnaire and all correspondence. Questionnaires were sent out to individuals with a covering letter explaining the purpose of the study and the use that the data obtained would be put to. Both Welsh and English language copies of these documents were sent. A two week response period was given. After this period, a further questionnaire and covering letter was sent to non-responders. The results of the questionnaire were then collated in Excel and analysed using SPSS.

### Comparative needs assessment

Comparative needs assessment compares levels and patterns of service provision in different geographical areas. We compared the current provision of bone densitometry services in North Wales with the rest of Wales and selected units in England and Scotland. Data included number of scanners, type of scanner used, staffing levels, cost of service, referral criteria, number of scans performed, and waiting times. Information on the location of DXA scanners in Wales was obtained from one of the unit managers.

### Unmet need for DXA scans in North Wales

The unmet need for DXA scans in North Wales was calculated using a widely accepted estimate of population requirements for DXA scanning of 1,000 per 100,000 population [14,15].

### Option appraisal of the DXA scanning service

An option appraisal and sensitivity analysis of 13 options for DXA scanning was conducted. Options considered included continuing the current provision via Gobowen, having a new static scanner based in Llandudno or elsewhere in North Wales, setting up a new mobile DXA scanning service, buying in scans from a mobile provider, and a combination of these options (Table 1).

**Table 1:** Options considered in Option Appraisal

**Option**	**Provider and location**
**Option 1**	Status Quo/do nothing
**Option 2**	Existing service with increased number of scans
**Option 3**	Existing service plus mobile scans purchased from another provider
**Option 4**	New service with static scanner at Llandudno only
**Option 5**	New service with static scanner at Llandudno plus existing service
**Option 6**	New service with static scanner at Llandudno plus mobile scans purchased from another provider
**Option 7**	New service with static scanner elsewhere in North Wales only
**Option 8**	New service with static scanner elsewhere in North Wales plus existing service
**Option 9**	New service with static scanner elsewhere in North Wales plus mobile scans purchased from another provider
**Option 10**	New service with mobile DXA scanner serving North Wales
**Option 11**	New service with mobile DXA scanner serving North Wales plus existing service
**Option 12**	New service with mobile DXA scanner with additional mobile scans purchased from another provider
**Option 13**	New service with mobile DXA scans purchased from another provider only

The table shows the 13 options considered in the option appraisal.

Limited criteria were agreed against which to value the options, including accessibility, GP practice opinion, user opinion, ease of staffing, ease of management, capacity, quality of environment and time to implement. These criteria were independently weighted by members of a DXA Advisory Group in order of importance and a mean weight was calculated for each criteria. The options were then scored against the criteria and assessed on a range of one to five as follows – 1-Very poor 2-Poor 3-Average 4-Good 5-Very good – to produce weighted benefit scores.

The capital, recurrent revenue, non-recurrent revenue and discounted costs for options 1–13 were next worked out. A Value for Money (VFM) score was calculated for all options by dividing the total discounted cost by the weighted benefit scores. The lower the score, the better the value for money.

A sensitivity analysis was performed to assess how sensitive the VFMs were to changes in the benefit criteria assigned, as there was some disagreement within the DXA Advisory Group as to the weights that should be allocated to particular criteria.

## Results

### Epidemiological needs assessment

We estimated that there are 7,000 men and 32,000 women with osteoporosis in North Wales. In 2002–3, there were 687 hip, 399 wrist & forearm and 308 vertebral osteoporotic fractures (Patient Episode Database Wales) [16] equating to approximately £5 M in hospital and £16 M in wider health & social costs [14]. Using PCA (prescribing cost analyses) data [17], it was estimated that North Wales spent £4.5 M on osteoporosis-related drugs in year studied, with 1.9 M of this on HRT and 1.8 M on bisphosphonates. This represents an 8% increase from the previous year compared with a 7% increase in Wales as a whole. North Wales spent about £46 on bisphosphonates (the most osteoporosis specific drug) per head of population estimated to have osteoporosis in 2002–3, compared with £37 across the rest of Wales.

### Corporate needs assessment

The surveys of general practices in North Wales (N = 121) and service users (N = 178) had response rates of 93% and 80% respectively, indicating the importance of this matter to the local area. Although the (current) service provided by Gobowen scored highly for satisfaction on both the general practice and user surveys, definite inequity of access to this service by area of residence was found. More than one quarter of practices in North Wales reported that they had had at least one patient in the last 12 months who had refused or been unable to travel to Gobowen because of travel distance. A number of practices also commented that their patients had difficulties in accessing Gobowen, especially the more at risk elderly population. Over half of the users who responded did not feel that Gobowen was conveniently located, 43% found it difficult to travel to the hospital for their scan and about the same number found it expensive. Over 90% of patients had travelled by car, 77% of them accompanied, with an average travel cost of £21. This equates to a £40,000 expenditure per annum on travel to Gobowen for North Wales residents.

Given a choice of where they would like a new DXA service to be located, practices chose a centrally located town (Llandudno) first, followed by 'somewhere else in North Wales', then a mobile service, with the existing service outside the region least favoured. There was variation in choice between the different LHBs with the western and central boards favouring Llandudno; and eastern boards preferring the current provider. This variation is shown in Table 2:

**Table 2:** Choice of location for future DXA service

**Local Health Board**	**Option 1: Existing DXA service in Gobowen**	**Option 2: New DXA service based at Llandudno Hospital**	**Option 3: New DXA service based elsewhere in N. Wales**	**Option 4: New Mobile DXA service covering all N. Wales**
**Anglesey**	4^th ^Choice	**1**^st^**Choice**	2^nd ^Choice	3^rd ^Choice
**Gwynedd**	4^th ^Choice	**1**^st^**Choice**	2^nd ^Choice	3^rd ^Choice
**Conwy**	4^th ^Choice	**1**^st^**Choice**	2^nd ^Choice	2^nd ^Choice
**Denbighshire**	4^th ^Choice	2^nd ^Choice	3^rd ^Choice	**1**^st^**Choice**
**Flintshire**	2^nd ^Choice	4^th ^Choice	**1**^st^**Choice**	3^rd ^Choice
**Wrexham**	**1**^st^**Choice**	4^th ^Choice	2^nd ^Choice	3^rd ^Choice
**North Wales**	3^rd ^Choice	**1**^st^**Choice**	2^nd ^Choice	4^th ^Choice

The table shows the overall choice of GP practices within the 6 LHBs in North Wales for the scanning options.

In contrast, the user sample chose the current provider first, followed by the central location, a mobile scanner and then 'another district hospital in North Wales'. However, about two-thirds of the sample chose a service other than the current provider. There was a significant difference in choice between users resident in the LHBs, with the western boards more likely to choose Llandudno and the eastern boards more likely to favour Gobowen. In interpreting this result, however, it needs to be noted that 40% of the Denbighshire user sample were from the south of the county, close to the existing service. User choices are shown in Table 3:

**Table 3:** User choice of future service options

**Local Health Board**	**Option 1: Existing DXA service in Gobowen**	**Option 2: New DXA service based at Llandudno Hospital**	**Option 3: New DXA service based 'elsewhere in N. Wales'**	**Option 4: New Mobile DXA service covering all N. Wales**
**Anglesey**	2^nd ^Choice	**1**^st^**Choice**	2^nd ^Choice	4^th ^Choice
**Gwynedd**	3^rd ^Choice	2^nd ^Choice	3^rd ^Choice	**1**^st^**Choice**
**Conwy**	3^rd ^Choice	**1**^st^**Choice**	2^nd ^Choice	4^th ^Choice
**Denbighshire**	**1**^st^**Choice**	3^rd ^Choice	4^th ^Choice	2^nd ^Choice
**Flintshire**	**1**^st^**Choice**	4^th ^Choice	2^nd ^Choice	2^nd ^Choice
**Wrexham**	**1**^st^**Choice**	4^th ^Choice	3^rd ^Choice	2^nd ^Choice
**North Wales**	**1**^st^**Choice**	2^nd ^Choice	4^th ^Choice	3^rd ^Choice

The table shows the overall choice of users resident within the 6 LHBs in North Wales for the scanning options.

### Comparative needs assessment

There are seven NHS DXA scanners in mid and south Wales. These performed 14,570 DXA scans in 2002–3 for a population of approximately 2,200,000, an overall rate of 660 scans/100,000 population, or 66% of the total required (assuming a population requirement for DXA scanning of 1,000 per 100,000 population).

### Unmet need for DXA scans in North Wales

It was estimated that North Wales requires approximately 6,500 scans per annum. The total number of central DXA scans (the gold standard diagnostic technique) provided to North Wales by the current provider in 2002–3 was 2058, giving a rate of DXA provision of 310 per 100,000 population, implying only 31% of the people in North Wales who meet the national guidelines were receiving central DXA scans. The greatest deficiencies in central DXA scanning provision were in Conwy and Denbighshire, which have some of the highest rates of hip fracture. There was also evidence of inequity of access to the current service by age and sex. 16% of referrals were in those less than 50, which is higher than one might have expected given the age-related burden of disease. In addition, fewer men were referred for bone densitometry than one might have expected given the burden of disease in this sex (10% of referrals were for men who made up 18.5% of those estimated to suffer from osteoporosis).

### Option appraisal of the bone densitometry service

The most suitable option identified was a DXA scanning service based in the central location of Llandudno. This option had the best VFM score in 15 out of the 16 sensitivity analyses and would improve the geographical accessibility for the majority of the residents of North Wales. It would also double the current central DXA scanning provision in the region (from 2,000 provided by Gobowen) to 4,000 provided by a scanner working full-time (expert opinion); this would lead to a scanning provision of about 60% of requirements, similar to the rest of Wales (66%). However, this option would not be able to provide the full complement of 6,500 scans and would reduce accessibility for the residents of Flintshire, Wrexham and South Denbighshire.

## Discussion

### Main findings

Our study found evidence of significant underprovision of bone densitometry services to the residents of North Wales. We also found definite inequity in access by geographical area of residence and evidence of inequity of access by age and sex.

### What is already known on the topic

There are a number of methods which can be used to inform and guide commissioning:

'Best Value' is a system which aims to help the public sector commission efficiently according to best value performance indicators (PVPIs). These are a statutory list of 90 indicators to measure local authority performance. Data is collected and audited annually by the Audit Commission [18].

Programme budgeting and Marginal Analysis(PBMA) is a method adopted and promoted by health economists. It sets out, in a systematic manner, patterns of resource use in health and social care by disease group, age band or service client group. It also helps to identify candidate services for expansion and contraction and the supporting evidence and criteria by which they may be compared and ranked, so as to increase benefits at the margin [19,20].

In health economics, multi attribute utility analysis has been used to measure individual's preferences for different health states [21]. It has also been used to generate health indices such as the Health Utilities Index [22]. The utility values gathered may be entered into economic models or used in cost effectiveness analysis.

### What this study adds

This paper is an example of a pragmatic needs assessment to inform evidence based commissioning. This study illustrates how a combined epidemiological, corporate and comparative approach to needs assessment provided the LHBs responsible for commissioning services across North Wales with objective, literature and user based evidence of the most suitable site for the DXA scanner. Such an approach offers all parties an objective basis on which to make and accept commissioning decisions. This study is generalisable to other groups of LHBs or PCTs in a similar setting.

### Limitations of this study

The questionnaires for professionals and service users may have introduced a potential bias as only a limited number of geographic options were presented.

This study is not a full economic evaluation alongside a clinical trial and as such cannot generate information as to the most efficient site for a DXA scanner [23]. However, the pragmatic, multi-objective approach takes into account local circumstances, current levels of provision, access issues and provided input into evidence based commissioning.

It may also be necessary to amend the results and conclusions of this study when the National Institute for Health and Clinical Excellence technology appraisal on the primary prevention of osteoporosis [24], the updated guidance on secondary prevention [25], plus the clinical guideline on osteoporosis [26], are published, as these may impact on the total number of scans required.

## Conclusion

The assessment identified significant unmet need for bone densitometry. A recommendation was made to improve access through the introduction of a new DXA scanning service based at Llandudno. This would double scanning provision provided and reduce travel costs and time for many North Wales residents. This recommendation was adopted by a joint commissioning group established by the six Local Health Boards in North Wales at the end of 2004 – evidence based commissioning in practice.

## Competing interests

The author(s) declare that they have no competing interests.

## Authors' contributions

RA managed the research project as part of his training in public health. SP, RR and ITR closely supervised the research project as educational and service tutors. DR provided statistical input and RTE provided health economics input. All the authors read and approved the final manuscript.
